# In‐Depth Extracorporeal Cardiopulmonary Resuscitation in Adult Out‐of‐Hospital Cardiac Arrest

**DOI:** 10.1161/JAHA.120.016521

**Published:** 2020-05-06

**Authors:** Mark Dennis, Sean Lal, Paul Forrest, Alistair Nichol, Lionel Lamhaut, Richard J. Totaro, Brian Burns, Claudio Sandroni

**Affiliations:** ^1^ Sydney Medical School University of Sydney Australia; ^2^ Greater Sydney Area Helicopter Emergency Medical Service New South Wales, Ambulance Service ??? Australia; ^3^ Istituto Anestesiologia e Rianimazione Università Cattolica del Sacro Cuore – Policlinico Universitario Agostino Gemelli – IRCCS Rome Italy; ^4^ University College Dublin‐Clinical Research Centre St Vincent’s University Hospital Dublin Ireland; ^5^ Department of Cardiology Royal Prince Alfred Hospital Sydney Australia; ^6^ Department of Anaesthesia Royal Prince Alfred Hospital Sydney Australia; ^7^ School of Public Health and Preventive Medicine Monash University Melbourne Australia; ^8^ Department of Intensive Care The Alfred Hospital Melbourne Australia; ^9^ Department of Intensive Care Royal Prince Alfred Hospital Sydney Australia; ^10^ INSERM U970 Team 4 “Sudden Death Expertise Center” Paris France; ^11^ Paris Descartes University Paris France; ^12^ SAMU de Paris‐DAR Necker University Hospital‐Assistance Public Hopitaux de Paris Paris France

**Keywords:** cardiac arrest, cardiopulmonary resuscitation, ECPR, extracorporeal circulation, Cardiopulmonary Arrest, Cardiopulmonary Resuscitation and Emergency Cardiac Care

## Abstract

The use of extracorporeal cardiopulmonary resuscitation (E‐CPR) for the treatment of patients with out‐of‐hospital cardiac arrest who do not respond to conventional cardiopulmonary resuscitation CPR) has increased significantly in the past 10 years, in response to case reports and observational studies reporting encouraging results. However, no randomized controlled trials comparing E‐CPR with conventional CPR have been published to date. The evidence from systematic reviews of the available observational studies is conflicting. The inclusion criteria for published E‐CPR studies are variable, but most commonly include witnessed arrest, immediate bystander CPR, an initial shockable rhythm, and an estimated time from CPR start to establishment of E‐CPR (low‐flow time) of <60 minutes. A shorter low‐flow time has been consistently associated with improved survival. In an effort to reduce low‐flow times, commencement of E‐CPR in the prehospital setting has been reported and is currently under investigation. The provision of an E‐CPR service, whether hospital based or prehospital, carries considerable cost and technical challenges. Despite increased adoption, many questions remain as to which patients will derive the most benefit from E‐CPR, when and where to implement E‐CPR, optimal post‐arrest E‐CPR care, and whether this complex invasive intervention is cost‐effective. Results of ongoing trials are awaited to determine whether E‐CPR improves survival when compared with conventional CPR.

**Nonstandard Abbreviations and Acronyms**

APACAR2A Comparative Study Between a Pre‐hospital and an In‐hospital Circulatory Support Strategy (ECMO) in Refractory Cardiac Arrest

BLENDER Blend to Limit Oxygen in ECMO: A Randomised Controlled Registry Trial

C‐CPR conventional cardiopulmonary resuscitation

CPR cardiopulmonary resuscitation

ECMO extracorporeal membrane oxygenation

E‐CPR extracorporeal cardiopulmonary resuscitation

EMS emergency medical service

EXACT Reduction of Oxygen After Cardiac Arrest trial

OHCA out‐of‐hospital cardiac arrest

OR odds ratio

RCA refractory cardiac arrest

ROSC return of spontaneous circulation

Sub 30 Feasibility of pre‐hospital ECPR implementation in under 30 minutes trial

TAME Targeted Therapeutic Mild Hypercapnia After Resuscitated Cardiac Arrest

Every year, ≈350 000 people in the United States^1^ and 275 000 in Europe^2^ experience an out‐of‐hospital cardiac arrest (OHCA). Overall survival to discharge from OHCA resuscitated with conventional cardiopulmonary resuscitation (C‐CPR) and advanced life support protocols including defibrillation, is reported to be between 2% and 15%.^1^, ^3^ These poor survival rates are consistent across geographical locations and have had only modest improvement over time.^4^

In patients with OHCA that is refractory to C‐CPR and in whom the cause of the OHCA is potentially reversible, extracorporeal cardiopulmonary resuscitation (E‐CPR) can provide a bridge to definitive treatment and recovery. The use of E‐CPR has increased 10‐fold in the past 10 years^5^ and many observational studies have shown encouragingly high survival rates.^6^, ^7^ However, no randomized controlled trials of E‐CPR versus C‐CPR have been published to date, thus the benefits of E‐CPR over C‐CPR remain unproven. This review examines the current literature on E‐CPR for OHCA, including patient selection, implementation models, post‐arrest care, cost‐effectiveness, and efficacy. We also highlight areas of ongoing research on E‐CPR and potential future developments.

## Extracorporeal Membrane Oxygenation and E‐CPR

Extracorporeal membrane oxygenation (ECMO) is a combination of a blood pump and an oxygenator that can be used to support either pulmonary or both pulmonary and cardiac function (venovenous or venoarterial configurations, respectively). E‐CPR is used to describe the use of venoarterial ECMO in refractory cardiac arrest (RCA). The increased adoption of E‐CPR has been facilitated by the advent of small, portable ECMO devices and circuit improvements. Attributable to advances in ECMO technology, it is possible to deliver either partial or full cardiorespiratory support for weeks or months, if required.

**Figure 1:**
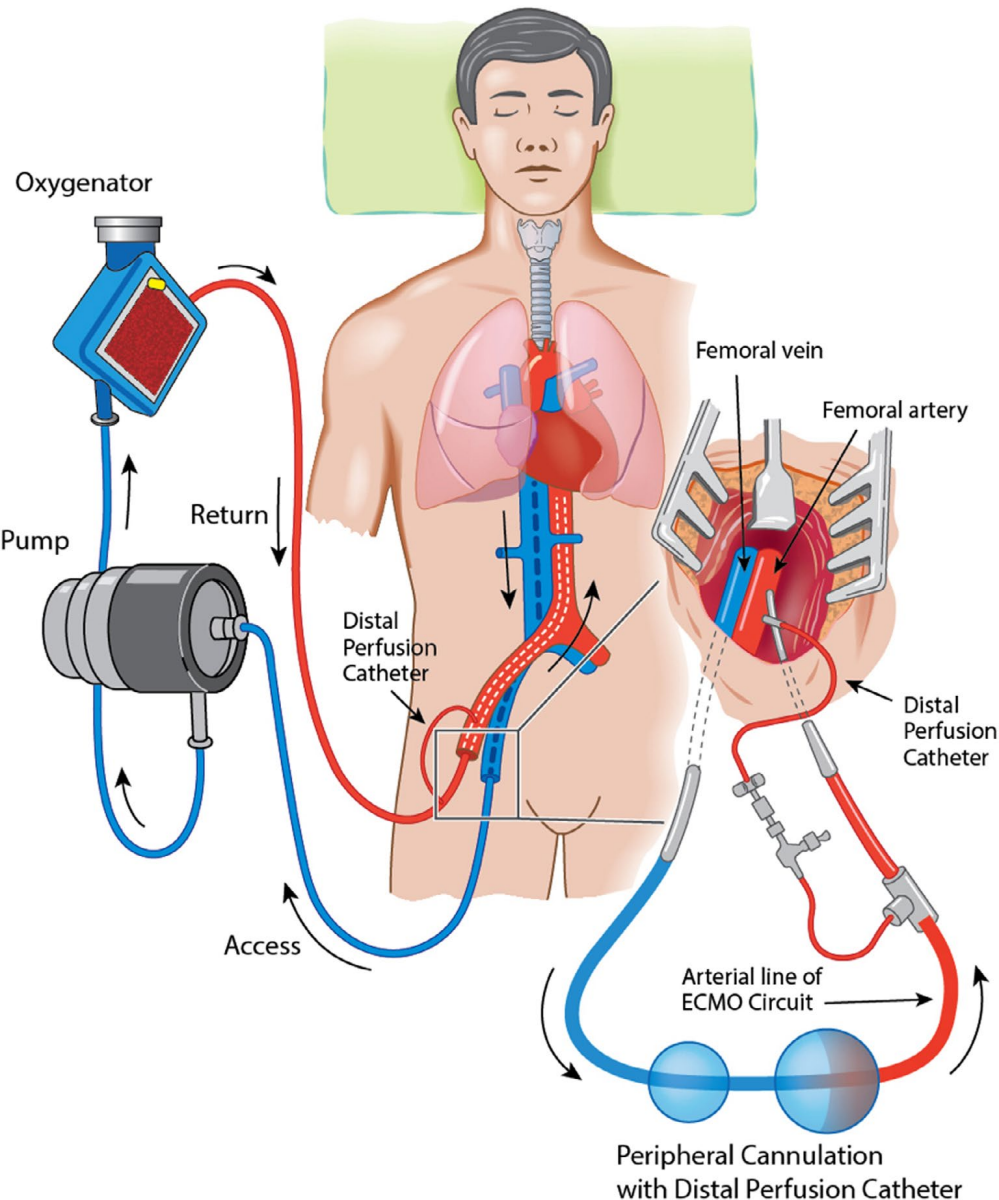
Venoarterial extracorporeal membrane oxygenation (ECMO) using a peripheral configuration. Venous blood (blue) is drained via a cannula positioned at the inferior vena cava to the right atrial junction and passes through the extracorporeal membrane where oxygenation and CO_2_ removal occurs. The now oxygenated blood (red) is returned via a “return” cannula position in the common iliac artery or descending aorta. The distal perfusion catheter, applied after ECMO support is established, is inserted into the superficial femoral artery distal to the insertion point of the femoral return cannula, and it supplies oxygenated blood to the distal limb to prevent distal limb ischemia.

**Figure 2:**
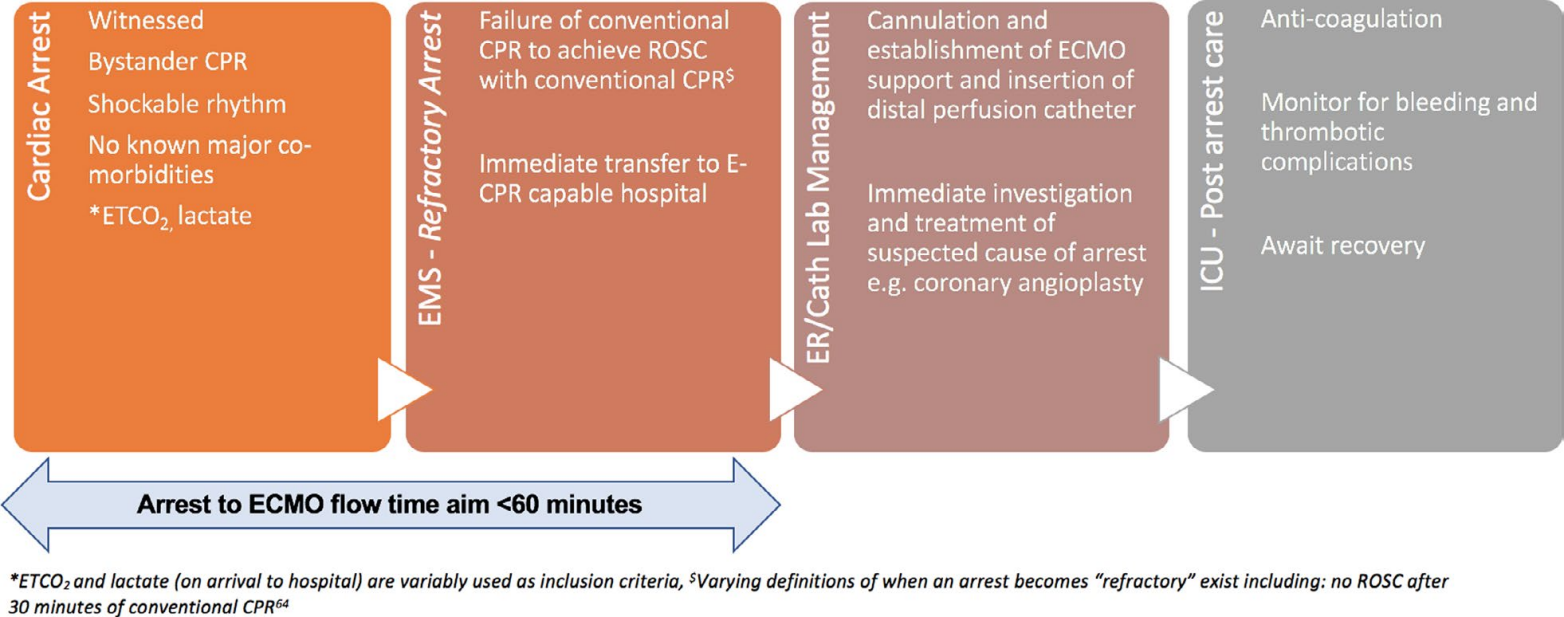
Key steps in extracorporeal cardiopulmonary resuscitation (E‐CPR) for out‐of‐hospital cardiac arrest (OHCA).^8^, ^9^ Key initial cardiac arrest variables of witnessed arrest, immediate bystander cardiopulmonary resuscitation (CPR) and shockable rhythm are currently required to qualify for a potential E‐CPR–eligible OHCA in most trials and services. If the arrest is refractory^8^, ^9^ to advanced life support measures and E‐CPR is available at a nearby hospital, the patient is transferred to the nearest E‐CPR–capable hospital. Many services utilize mechanical CPR to enable ongoing chest compression during transfer. Prearrival notification to the accepting hospital by emergency medical services (EMS) is often made. On arrival to the hospital, cannulation, establishment of extracorporeal membrane oxygenation, and subsequent definitive treatment of the underlying cause of the arrest is made. Standard postcardiac arrest care is implemented in the intensive care unit (ICU). *End‐tidal CO_2_ (ETCO_2_) and lactate (on arrival to hospital) are variably used as inclusion criteria. ^$^Varying definitions of when an arrest becomes “refractory” exist including no return of spontaneous circulation (ROSC) after 30 minutes of conventional CPR.^9^

An ECMO circuit consists of a centrifugal pump and a membrane oxygenator for oxygen delivery, CO_2_ removal, and temperature management. In E‐CPR, venoarterial ECMO is established while cardiopulmonary resuscitation (CPR) is ongoing. The drainage (access) cannula is placed into the inferior vena cava via the femoral vein, and the “return” cannula is inserted into the femoral artery to the level of the common iliac artery (Figure 1). E‐CPR for OHCA is usually provided as part of a “bundle of care,”^8^, ^9^ which includes early patient transfer to hospital, mechanical CPR to provide effective CPR during transport, early initiation of E‐CPR, and early definitive treatment (eg, coronary angioplasty [Figure 2]).

## Rationale for E‐CPR

C‐CPR either by manual or mechanical chest compression is the mainstay of circulatory support during cardiac arrest. However, even with optimal technique, C‐CPR delivers only 15% to 25% of normal cardiac output^10^ (or a cardiac index of ≈0.6 L/min per m^2^)^11^ with consequent rapid development of ischemic damage to vital organs (this is commonly referred to as a *low‐flow* state). Conversely, E‐CPR can provide near‐normal levels of cerebral and end organ perfusion.^12^ The ability to provide full cerebral and end organ blood supply, even for days or weeks, with E‐CPR has enabled a paradigm shift in cardiac arrest—preservation of the brain while awaiting return of spontaneous circulation (ROSC), definitive care, and cardiac recovery.

## Indications and Patient Selection for E‐CPR

To date, there are no unanimously accepted indications for E‐CPR. The consensus‐based French Ministry of Health guidelines^13^ recommend considering E‐CPR for RCA when ≥1 of the following conditions are met: (1) known reversible cause (eg, drug intoxication or hypothermia), likely to require prolonged life support; (2) signs of life during CPR; (3) no‐flow (the period of time from cardiac arrest to the commencement of CPR) and low‐flow durations <5 and 100 minutes, respectively; (3) an initial shockable rhythm; and (4) an end‐tidal CO_2_ >10 mm Hg. However, these guidelines have not been prospectively validated. Moreover, few subsequent studies on E‐CPR for OHCA have adopted all of these indications and some have adopted none of them.^14^

The inclusion criteria used in many E‐CPR studies include age 18 to 65 years or 18 to 70 years, witnessed RCA, immediate bystander CPR, initial shockable rhythm, access to immediate coronary angiography, and an anticipated low‐flow period (ie, the interval from C‐CPR commencement to E‐CPR) of <60 minutes.^8^

A systematic review and meta‐analysis of 841 E‐CPR–treated patients with OHCA (Debaty et al^15^) for prognostic factors or neurological outcome and survival demonstrated that an initial shockable rhythm was associated with twice the odds (odds ratio [OR], 2.20; 95% CI, 1.30–3.72) of favorable outcomes (survival with good neurological function) than nonshockable rhythms. Other factors associated with favorable outcomes were low‐flow duration (54 versus 64 minutes) (OR, 0.90; 95% CI, 0.81–0.99 [*P*=0.04], higher arterial pH (difference, 0.12; 95% CI, 0.03–0.22 [*P*=0.01], and lower serum lactate (difference, −3.52 mmol/L; 95% CI, −5.05 to −1.99 [*P*<0.001]). Bystander CPR was also associated with higher summary odds for favorable outcome (OR, 2.81; 95% CI 0.95–8.32), although this was not statistically significant.

Age has been variably reported as a negative prognostic marker for E‐CPR. Goto et al^16^ found that age older than 70 years was associated with poor outcomes. However, Yu et al^17^ found that age older than 75 years was not predictive of poor outcome if the low‐flow duration was <60 minutes. Debaty et al^15^ also did not find an association between adverse prognosis and advancing age.^15^ Although age is not currently considered a contraindication to E‐CPR, most studies report a median age of patients undergoing E‐CPR younger than 60 years^14^—lower than the median age of 64 years reported in the largest OHCA registries on adult OHCA.^18^

Other common inclusion criteria for E‐CPR include signs of life and end‐tidal CO_2_ level >10 mm Hg on arrival to the emergency department.^7^, ^19^ Their value as inclusion criteria in E‐CPR is yet to be systematically assessed.

## When Should the Transition to E‐CPR Occur?

The optimal time point to transition from C‐CPR to E‐CPR strategy is yet to be determined. Implementation of E‐CPR too early may unnecessarily expose patients with C‐CPR in whom ROSC may potentially occur to an expensive procedure with significant additional risks, whereas delaying E‐CPR implementation may jeopardize the potential benefit from the intervention by increasing the risk of critical brain and end organ injury from prolonged hypoperfusion.

After more than 35 minutes of C‐CPR without subsequent E‐CPR, <1% of patients with OHCA overall will have spontaneous ROSC and survive with a favorable neurological outcome.^20^ Therefore the decision to commence E‐CPR needs to occur before this point. In an analysis of North American emergency medical services (EMS) data,^21^ transport to an E‐CPR center between 8 to 24 minutes of EMS resuscitation had the highest sensitivity and specificity for favorable outcome, with 16 minutes being the optimal time.^21^ In a retrospective Korean propensity‐matched analysis (444 patients with C‐CPR and 55 patients with E‐CPR), Kim et al^22^ reported that E‐CPR outcomes became superior to C‐CPR after 21 minutes of resuscitation. Additionally, E‐CPR was associated with improved neurologically intact survival from RCA (21% versus 0% for 41–60 minutes of C‐CPR). These data suggest that E‐CPR may be considered after 10 to 20 minutes of unsuccessful C‐CPR^8^, ^23^ in selected patients.^8^, ^24^ This, however, presents significant logistical challenges. In order to meet these targets, EMS teams may need to alter their protocols to facilitate shorter on‐scene times and adopt mechanical C‐CPR to facilitate effective CPR during transportation, ie, moving from a “stay and treat” to a “load and go” strategy. An alternative is implementation of E‐CPR at the site of the cardiac arrest—*prehospital E‐CPR—*which is discussed below. Any of these options would represent a major change for many EMS systems.

## E‐CPR Versus C‐CPR

While no published randomized control trials of E‐CPR versus C‐CPR have been published to date, a small number of propensity‐matched observational studies have been completed. In a study of 162 adult patients (E‐CPR=53, C‐CPR=109)^25^ with witnessed cardiac arrest of cardiac origin who had undergone CPR for longer than 20 minutes, the intact survival rate was higher in the E‐CPR group than in the propensity‐matched C‐CPR group (29.2% [7/24] versus 8.3% [2/24], log‐rank *P*=0.018). The Save‐J (Study of Advanced Cardiac Life Support for Ventricular Fibrillation With Extracorporeal Circulation in Japan)^26^ prospectively compared E‐CPR and C‐CPR outcomes in 454 patients with OHCA who had an initial shockable rhythm and no ROSC until at least 15 minutes after arrival to the hospital. Favorable neurological outcome at 6 months was found in 29 of 260 patients with E‐CPR (11.2%) versus 5 of 194 patients (2.6%) in the C‐CPR group (*P*=0.001).

Most recently, a large Parisian registry^27^ compared C‐CPR– and E‐CPR–treated patients with OHCA. Overall survival was 8.4% in 525 patients treated with E‐CPR and 8.6% in 12 666 patients treated with C‐CPR (*P*=0.91). After multivariable regression analysis, the provision of E‐CPR was not associated with increased survival (OR, 1.3; 95% CI, 0.8–2.1 [*P*=0.24]). A propensity score–matched analysis of 429 pairs for survival adjusted for major confounders did not find a significant difference in survival between the C‐CPR and E‐CPR groups (OR, 0.8; 95% CI, 0.5–1.3 [*P*=0.41]). However, despite the large size of that study, several limitations exist. First, there was no specific time to transition to E‐CPR strategy nor strict inclusion criteria for E‐CPR. This resulted in only 27% of patients having a shockable rhythm and 39% of E‐CPR–treated patients receiving CPR for over 90 minutes. Further, E‐CPR was initiated at the discretion of treating clinicians, thereby introducing selection bias by known or unknown confounders, which may not have been corrected for in propensity and regression analysis.

Bartos et al^28^ retrospectively compared 160 consecutive patients with OHCA with refractory ventricular tachycardia/ventricular fibrillation treated with E‐CPR as per predefined University of Minnesota protocol^29^ with 654 patients who received standard CPR as part of the Amiodarone, Lidocaine or Placebo in Out‐of‐Hospital Cardiac Arrest Study (ALPS)^30^ using multivariable analysis. Despite E‐CPR–treated patients having longer mean duration of CPR (60 versus 35 minutes, *P*<0.001), the E‐CPR cohort was associated with better neurological outcomes at all CPR durations <60 minutes (33% E‐CPR group versus 23%, *P*=0.01). No patients treated with C‐CPR survived if their period of resuscitation was >40 minutes compared with 25% (9/26) in the E‐CPR group.

While some systematic reviews and meta‐analyses suggested that E‐CPR may improve survival or neurological outcome^31^, ^32^, ^33^, ^34^, ^35^, ^36^ the most recent and comprehensive systematic review from Holmberg et al^14^ yielded mixed results. This review included 12 studies on adult OHCA reporting survival to discharge. Of these, 7 showed higher odds of survival with E‐CPR versus C‐CPR, while 5 showed lower odds. Among 8 studies reporting survival with good neurological outcome, 7 favored E‐CPR. A meta‐analysis was not performed because of high study heterogeneity.

All systematic reviews of E‐CPR have rated the quality of evidence as very low. The inability of the available reviews to draw consistent strong conclusions on E‐CPR for OHCA is a direct result of significant limitations of the individual studies reviewed. Most reviewed studies were small, single‐center, and from diverse locations with different EMS systems, all of which limit the quality of the comparative data. Furthermore, inclusion criteria for E‐CPR differ between studies, and other unmeasured confounders may limit the internal and external validity of results. Finally, several E‐CPR studies have assessed a bundle of care that includes expedited transport to hospital, mechanical CPR, and immediate coronary angiography. While these have not been shown to be convincingly beneficial in their respective own right,^37^, ^38^, ^39^ it is possible that a whole E‐CPR “bundle” contributes more to outcomes than E‐CPR in isolation.

Based on the low quality of the current E‐CPR literature, in its recent Focused Update on Advanced Cardiovascular Life Support,^40^ the American Heart Association concluded that there was insufficient evidence to recommend the routine use of E‐CPR but that it should be considered as rescue therapy for selected patients when it can be rapidly implemented and supported by skilled providers (class 2b, level of evidence C).

### Prehospital E‐CPR

Implementation of E‐CPR at the cardiac arrest scene could potentially decrease the time to E‐CPR initiation. In a cohort study from Paris,^19^ two periods of different E‐CPR management strategies were compared. The first period (n=114 patients) included a mandatory 30‐minute interval of C‐CPR before either transport to hospital (if within 20 minutes range) or initiation of prehospital E‐CPR. With this strategy, low‐flow duration was 93±27 minutes, with 8% neurologically intact survival. In the second period (n=42 patients), a more aggressive prehospital E‐CPR strategy and more defined inclusion criteria were implemented, with a dedicated on‐call E‐CPR team that was dispatched to all patients with OHCA younger than 70 years. Attempts to perform E‐CPR commenced after 20 minutes of unsuccessful C‐CPR, with the aim of initiating pump flow within 60 minutes from the onset of cardiac arrest. After the implementation of this strategy, the mean low‐flow interval was reduced by 20 minutes and neurologically intact survival improved to 29% (21% absolute increase, *P*<0.001). In the propensity‐matched analysis of the 2 cohorts, prehospital E‐CPR was associated with significantly reduced low‐flow duration and higher rates of ROSC compared with hospital‐based E‐CPR, even if it was not an independent predictor of survival to discharge.

There are several prospective studies (either in progress or planned) to assess the feasibility and efficacy of prehospital E‐CPR. For example, the London Sub 30 (Feasibility Study of a Pre‐Hospital Extra‐Corporeal Membrane Oxygenation (ECMO) Capable Advanced Resuscitation Team at Achieving Blood Flow Within 30 Minutes in Patients With Refractory Cardiac Arrest) study (NCT03700125)^19^ will attempt to establish E‐CPR within 30 minutes of OHCA using mobile prehospital E‐CPR teams in London. The ongoing Paris APACAR2 (A Comparative Study Between a Pre‐hospital and an In‐hospital Circulatory Support Strategy (ECMO) in Refractory Cardiac Arrest) (NCT02527031)^41^ is randomizing patients with OHCA to receive either prehospital or hospital E‐CPR, depending on their location and predicted transport time to hospital. Patients with prehospital E‐CPR will receive E‐CPR between 20 and 30 minutes of C‐CPR. Participants randomized to hospital‐based E‐CPR will be transferred to the hospital with mechanical CPR. Selection criteria include no‐flow duration <5 minutes, age 18 to 65 years, refractory arrest (defined as 20 minutes of C‐CPR), and either shockable rhythm or signs of life during resuscitation.

## Post–E‐CPR Resuscitation Care

Intensive care management of patients with E‐CPR is complex and involves a multidisciplinary approach. There are few data on how patients with E‐CPR should be managed, and conventional postarrest protocols are generally utilized. The optimal management of arterial oxygen (partial pressure of O_2_) and CO_2_ (partial pressure of CO_2_) post‐OHCA has not been identified in patients with C‐CPR or patients with E‐CPR. Arterial hyperoxia is common postcardiac arrest (with or without E‐CPR) and has been associated with unfavorable outcomes in some observational C‐CPR studies.^42^, ^43^, ^44^

Following the initiation of other forms of ECMO, a rapid reduction in partial pressure of CO_2_ has been associated with adverse neurological outcomes.^45^ It has been postulated that this could be caused by an acute reduction in cerebral blood flow.^45^ However, in a randomized study^46^ of patients with C‐CPR and ROSC comparing low‐normal versus high‐normal partial pressure of CO_2_ and normoxia versus moderate hyperoxia, no significant difference in levels of neurological biomarkers was found.^46^ This study was underpowered to identify differences in clinical outcomes.

The randomized controlled trials of reduction of oxygen administration to target an oxygen saturation of 90% to 94%, compared with 98% to 100%, as soon as feasible following successful resuscitation from OHCA (EXACT [Reduction of Oxygen After Cardiac Arrest trial]—NCT03138005) and of oxygen management in patients with ECMO (BLENDER [Blend to Limit Oxygen in ECMO: A Randomised Controlled Registry Trial]—NCT03841084), comparing clinical outcomes in patients with ECMO randomized to receive either 100% oxygen to the oxygenator versus a restrictive strategy, as well as the upcoming TAME [Targeted Therapeutic Mild Hypercapnia After Resuscitated Cardiac Arrest] study (NCT03114033) comparing mild hypercapnia versus normocapnia in C‐CPR OHCA, may help guide future CO_2_ and oxygen management.

In patients undergoing E‐CPR, systemic anticoagulation is required to prevent thrombotic complications from the patient–circuit interface. However, this increases the risk of bleeding, and in patients with E‐CPR, significant bleeding has been reported in up to 70% of cases and is associated with worse outcomes.^47^ Unfractionated heparin is the most commonly used anticoagulant during ECMO. This is routinely monitored using either activated clotting time, partial thromboplastin time, or anti‐Xa activity, the former 2 of which can be measured as point‐of‐care tests.^48^, ^49^ More recently, thromboelastography and thromboelastometry (rotational thromboelastometry) have been investigated as alternative tests for monitoring anticoagulation during ECMO. Unlike activated partial thromboplastin time, which is plasma‐based, rotational thromboelastometry and thromboelastography are point‐of‐care viscoelastic tests that assess multiple coagulation functions in whole blood, including platelet function, fibrinogen function, and fibrinolysis. Preliminary evidence indicates that viscoelastic tests are feasible for anticoagulation management during extracorporeal circulation and are associated with reduced heparin consumption compared with activated partial thromboplastin time–based protocols.^50^ Thrombin inhibitors (such as bivalirudin) are mainly used when heparin is contraindicated or, as low antithrombin 3 levels are common on ECMO,^51^ in heparin resistance and may enable more tightly controlled anticoagulation^52^ in patients undergoing extracorporeal circulation.

## Cost‐Effectiveness of E‐CPR

Significant training, skill, equipment, and expense is required to provide an effective E‐CPR service. Three recent studies from North America, Australia, and Japan (Table S1) reported that E‐CPR was within accepted cost thresholds for advanced medical interventions.^53^, ^54^, ^55^ These costs ranged between $16 000 to $52 000/quality‐adjusted life‐years, depending on geographical location. E‐CPR may be cost‐effective for several reasons; patients with E‐CPR are generally younger than patients with C‐CPR and have good neurological outcomes with minimal residual disabilities and long‐life expectancy postevent. In these studies, however, the maximum follow‐up period was only 1 year,^55^ and long‐term outcome data are needed in future cost‐effectiveness analyses. The cost‐effectiveness of a prehospital E‐CPR strategy is unknown.

The rates of death by neurological criteria in patients resuscitated with E‐CPR are ≈3 times higher than in those resuscitated with C‐CPR, resulting in an increased potential for organ donation.^56^, ^57^ As of yet, the potential for increased organ donation has not been considered in cost‐effectiveness modelling of E‐CPR programs and presents some ethical questions.

## Future Directions of E‐CPR

Using most commonly used inclusion criteria (ie, aged 18 to 65 years, witnessed cardiac arrest, CPR started within 10 minutes, absence of asystole as the first cardiac rhythm on EMS arrival), it is estimated that only 4% to 11%^58^, ^59^, ^60^ of all patients with OHCA would be potentially eligible for E‐CPR.^60^ The relative infrequency of RCAs meeting current E‐CPR inclusion criteria (even in tertiary referral centers)^27^, ^59^, ^60^ inherently limits large sample cohorts and therefore the ability to accurately plan recruitment timelines. Moreover, given the known poor outcomes with extended C‐CPR, with neurologically intact outcomes of <1% at ≈35 to 40 minutes,^20^, ^61^ some clinicians have raised ethical concerns regarding randomization of patients to continued C‐CPR (when it may already be deemed to have failed) and E‐CPR is available. However, a small number of randomized controlled trials are currently comparing E‐CPR with C‐CPR (Table).^62^, ^63^, ^64^, ^65^

**Table 1:** Upcoming Randomized Controlled Trials on E‐CPR

Trial Name and Registration Number	Sample Size	Location	Inclusion Criteria	Estimated Completion
INCEPTION (NCT03101787)^62^ Standard arm: standard treatment as per ERC guidelines. Intervention*:* preclinical resuscitation by EMS and transport to the ED with ongoing mechanical cardiopulmonary resuscitation. Initiation of E‐CPR in the ED	110	The Netherlands	Age 18–70 y Witnessed OHCA Initial rhythm of ventricular fibrillation or ventricular tachycardia when external defibrillation administered Bystander CPR No ROSC within 15 min of C‐CPR	July 2021
EROCA (NCT03065647^63^ Standard arm*:* BLS and ACLS by EMS per existing protocols at the scene of the cardiac arrest. Intervention arm*:* patients with OHCA refractory to initial BLS and ACLS will be transported by EMS with ongoing mechanical CPR and ACLS to an ED capable of initiating E‐CPR. E‐CPR instituted within ED	30	Michigan, United States	Age 18–70 y OHCA of presumed nontraumatic cause Predicted arrival time at E‐CPR–capable hospital within 1 h Witnessed arrest or initial shockable rhythm Persistent cardiac arrest after initial cardiac rhythm analysis and shock	December 2020
Prague OHCA Study (NCT01511666)^64^ Standard arm*:* patients in the standard arm will be further managed as per ERC guidelines. Coronary angiography will be performed only if indicated according to routine practice, and mild therapeutic hypothermia will be instituted as soon as possible as per guidelines. Intervention arm: (hyperinvasive) immediate institution of a mechanical chest compression device and prehospital intra‐arrest cooling by RhinoChill device (BeneChill). Directly transferred to cardiac center catheterization laboratory under continuous CPR. After admission to cardiac catheterization laboratory for coronary angiography +/− E‐CPR	170	Prague, Czech Republic	Age 18–65 y Witnessed OHCA of presumed cardiac cause Minimum of 5 min of advanced life support performed by emergency medical team without sustained ROSC ECMO team and bed‐capacity in cardiac center available	December 2020
APACAR2 (NCT02527031)^65^ Patients with refractory OHCA—defined by the failure of EMS to resuscitate at the 20th min of cardiac arrest with a minimum of 3 automatic external defibrillations or equivalent analysis will be randomized to prehospital arm: E‐CPR in prehospital setting—implementation of E‐CPR support at site of cardiac arrest and then transfer to hospital; or in‐hospital arm: transfer to hospital for E‐CPR implementation in hospital setting	210	Paris, France	Adults older than 18 y and those younger than 65 y Refractory cardiac arrest (defined by the failure of professionals to resuscitate at the 20th min of cardiac arrest with a minimum of 3 defibrillator shocks Beginning of C‐CPR within the first 5 min after cardiac arrest (no‐flow duration <5 min) with shockable rhythm or the presence of signs of life during resuscitation (any rhythm) Medical cause of the cardiac arrest End‐tidal CO_2_ >10 mm Hg at the time of inclusion Absence of major comorbidities and E‐CPR team available on‐site within 40 min of cardiac arrest	March 2020

ACLS indicates advanced cardiac life support; APACAR2, A Comparative Study Between a Pre‐hospital and an In‐hospital Circulatory Support Strategy (ECMO) in Refractory Cardiac Arrest; BLS, basic life support; CPR, cardiopulmonary resuscitation; C‐CPR, conventional cardiopulmonary resuscitation; E‐CPR, extracorporeal cardiopulmonary resuscitation; ED, emergency department; EMS, emergency medical services; ERC, European Resuscitation Council; EROCA, E‐CPR for Refractory Out‐Of‐Hospital Cardiac Arrest; INCEPTION, Early Initiation of Extracorporeal Life Support in Refractory OHCA; OHCA, out‐of‐hospital cardiac arrest; and ROSC, return of spontaneous circulation.

Although difficult to conduct, large‐scale multicenter studies of E‐CPR are needed to address the significant uncertainties regarding efficacy, case selection, timing, and prognostic markers versus C‐CPR. In future studies, standardized definitions of key variables including what constitutes an RCA and CPR duration or arrest to ECMO flow times is required to enable more robust comparisons of outcomes. Moreover, documentation and adjustment for other known prognostic variables including precardiac arrest performance status and intracardiac arrest variables (eg, end‐tidal CO_2_, lactate, pH, and potassium) is required.^14^

An initial shockable rhythm is a common entry criterion for most E‐CPR programs. This is present in only about one third of OHCA cases^66^ and has a significantly better outcome than nonshockable rhythms. However, it is possible that patients with initial nonshockable rhythms may also benefit from E‐CPR if other favorable prognostic markers are present.^67^

Should E‐CPR be proven to improve survival in selected patients with OHCA, there would be significant organizational and economic implications for health systems to ensure optimal equity of access.

High‐volume venovenous ECMO centers have better outcomes than low‐volume centers.^68^ Given the high complexity of patients with E‐CPR, managing them in major centers with significant experience is reasonable. A composite model of having hospital centers that establish E‐CPR support, then transfer the patient to a larger center for continued care, has been proposed in a “hub and spoke”–type format. However, this model is yet to be tested in any large‐scale settings and may require additional resources to facilitate the transfer of critically unwell patients with E‐CPR between locations.

## Conclusions

E‐CPR for refractory OHCA may improve outcomes in carefully selected patients. However, the evidence base for E‐CPR efficacy, patient selection, and postcardiac arrest management is poor. Much work is needed in this area and large‐scale high‐quality trials, although challenging, should be made a priority.

## Disclosures

None.

## Supporting information


**Table S1 References 54–56**
