# Supplementary material for: In‐Depth Extracorporeal Cardiopulmonary Resuscitation in Adult Out‐of‐Hospital Cardiac Arrest
Source: J Am Heart Assoc. 2020 May 6;9(10):e016521. doi: 10.1161/JAHA.120.016521 (PMC7660839; doi:10.1161/JAHA.120.016521)
Supplement: Supplementary file 1 — Table S1 References 54–56 [file JAH3-9-e016521-s001.pdf]

SUPPLEMENTAL MATERIAL

Table S1. Summary of E-CPR cost effectiveness studies.

	Location	Pt No.	Cost per ECPR patient (USD) to hospital discharge or death	Cost per survivor*	Cost per QALY	ICER per QALY
Bharmal et al[56]	North America	32	$125,683 (IQR $49,751-$206,341)	NA	$56,156	NA
Dennis et al[54]	Australia	62	$52,615 (SD $53, 016)	$88,167	NA	$17,648
Kawashima et al[55]	Japan	120	$39,633 VT/VF, $35,609 ASYS/PEA	$213,656 PEA/ASYS, $101,669 VT/VF	$11,081 VT/VF, $29,447 ASYS/PEA	$16,246

**Cost per survivor calculated as total hospital cost of all patients divided by number of survivors*
